# Electrostatic Repulsion Governs TDP-43 C-terminal Domain Aggregation

**DOI:** 10.1371/journal.pbio.1002447

**Published:** 2016-04-20

**Authors:** Miguel Mompeán, Avijit Chakrabartty, Emanuele Buratti, Douglas V. Laurents

**Affiliations:** 1 Instituto de Química Física “Rocasolano” CSIC, Madrid, Spain; 2 Department of Biochemistry, University of Toronto, Toronto Medical Discovery Tower 4–305, MaRS Centre, Toronto, Canada; 3 International Centre for Genetic Engineering and Biotechnology, Trieste, Italy; Brandeis University, UNITED STATES

## Abstract

TDP-43 is a protein that forms aggregates implicated in amyotrophic lateral sclerosis. In response to a recent study, this Formal Comment argues that the pH-dependent solubility of this protein is better explained by the mutual repulsion of charged groups than by the formation of hydrogen bonds.

TDP-43 is a protein with multiple crucial functions in RNA processing and mRNA-protein (mRNP) particle formation, two processes that are strongly implicated in amyotrophic lateral sclerosis (ALS) and frontotemporal lobar dementia (FTLD) [1]. The recently published structural characterization by Lim, Wei, Lu, and Song [2] of the complete (residues 262–414) C-terminal domain (CTD) of TDP-43 and three pathologically relevant variants, and the interaction of this region with membrane-mimetics and DNA oligos, builds on our characterization of a smaller Q/N-rich C-terminal segment (residues 341–367) [3,4]. Together, these studies mark a significant advance in our understanding of this important protein. In particular, the results by Lim, Wei, Lu, and Song [2] show that the CTD exists as an intrinsically disordered conformational ensemble, which can be nudged by certain conditions into forming a “hydrogel” (a putatively functional amyloid) or coerced by mutation into adopting hypothetically pathological amyloid structures. The ability of membrane-mimetics to induce helix formation in a hydrophobic segment of TDP-43's CTD and to increase the aggregation rate suggests a link between membrane interaction and pathological cytotoxicity.

Nevertheless, the interpretation of some of the data could be improved by additional considerations that we would like to address specifically in this comment. In particular, based on the ability of the TDP-43 CTD to interact with membrane-mimetics, the authors claim that membrane disruption could be a disease mechanism. This assertion is plausible in light of results obtained for other aberrant polypeptide aggregates implicated in Alzheimer's disease and type 2 diabetes (as cited by Song and coworkers). However, there are literally thousands of proteins that are known to interact with membranes without disrupting them. For this reason, it is crucial to carry out well-established tests already used for short CTD fragments [5] to determine if aggregates of the complete CTD of TDP-43 can disrupt membranes.

One singularly perplexing proposal of Song and coworkers involves the strong pH dependence of the CTD’s aggregation and amyloid formation. In our opinion, Lim et al. [2] strike a false chord when they propose that

“…in the TDP-43 prion-like domain at pH 4.0, most backbone amyloid protons are involved in forming hydrogen bond networks with side chain oxygen atoms…”

(page 14, second paragraph)

to account for the lack of aggregation and amyloid formation at pH 4.0. They also claim that these interactions would be intramolecular and break down at pH 6.8, leading to faster aggregation. However, this assertion seems to be at odds with the generally very small changes observed in the circular dichroism (CD) spectra and nuclear magnetic resonance (NMR) temperature coefficients over this pH range (see SF1 and SF6, ref. [2]). Furthermore, no mechanism is advanced to explain how these H-bonds weaken in a pH-dependent manner. Finally, this assertion is untenable considering that there are only 60 side chain oxygen atoms in the CTD, a number insufficient to form H-bonds to the majority of the 152 backbone HN groups as claimed.

In fact, regarding this specific issue, we believe that there is a simpler and more logical explanation for their observation.

The solubility of folded proteins has long been known to vary with the pH and is typically lowest at pH values at which the protein carries a small net charge [6]. The solubility increases at those pH values at which the protein molecules present a higher net charge (positive or negative). This idea is supported by results of protein variants carrying multiple charge substitution mutations (Fig 1A) [7]. Moreover, related results have been obtained for Aβ and a series of variants carrying charge substitution mutations [8]; namely, aggregation and amyloid formation depend on the proximity to each polypeptide's isoelectric point (Fig 1B). These results are especially pertinent, since Aβ, like the CTD of TDP-43, is an intrinsically disordered polypeptide with a marked tendency to form amyloid.

**Fig 1:**
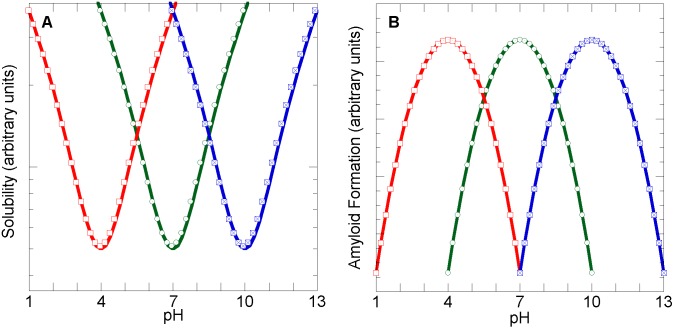
Protein aggregation and amyloid formation are favored by a low net charge. Idealized dependence of the solubility (**A**) or amyloid formation (**B**) by proteins with isoelectric points at pH 4 (red), 7 (green), and 10 (blue). Adapted from Fig 15–2 of [6] and Fig 1 of [7] (panel **A**) and Fig 1 of [8] (panel **B**). Please see S1 Data, columns A–E and columns G–J for the underlying data of panels A and B, respectively.

Bearing these considerations in mind, we have calculated the net charge of the CTD of TDP-43 as a function of pH on the basis of its‎ content of charged termini and residues (1D, 2E, 7H, 1Y, 2K, and 5R) and the reported pKa values for disordered chains (Fig 2A, S1 Data) [9]. The net charge on this CTD construct is high (+12.1) at pH 4, but decreases modestly (to +10.3) by pH 5.0 as the carboxylate groups acquire negative charge, and decreases significantly at pH 6.8 (to +6.0), as the seven imidazolium groups in His264 and the C-terminal hexaHis tag residues lose their positive charges. Strikingly, in fact, this decreased net charge parallels the increased aggregation observed by Lim, Wei, Lu, and Song as the pH increases from 4.0 to 5.0 to 6.8. Therefore, we conclude that the pH dependence of amyloid formation by the CTD is governed by electrostatic repulsion (Fig 2B), in accord with previous results on Aβ and its charge substitution variants (Fig 1 in [8]).

**Fig 2:**
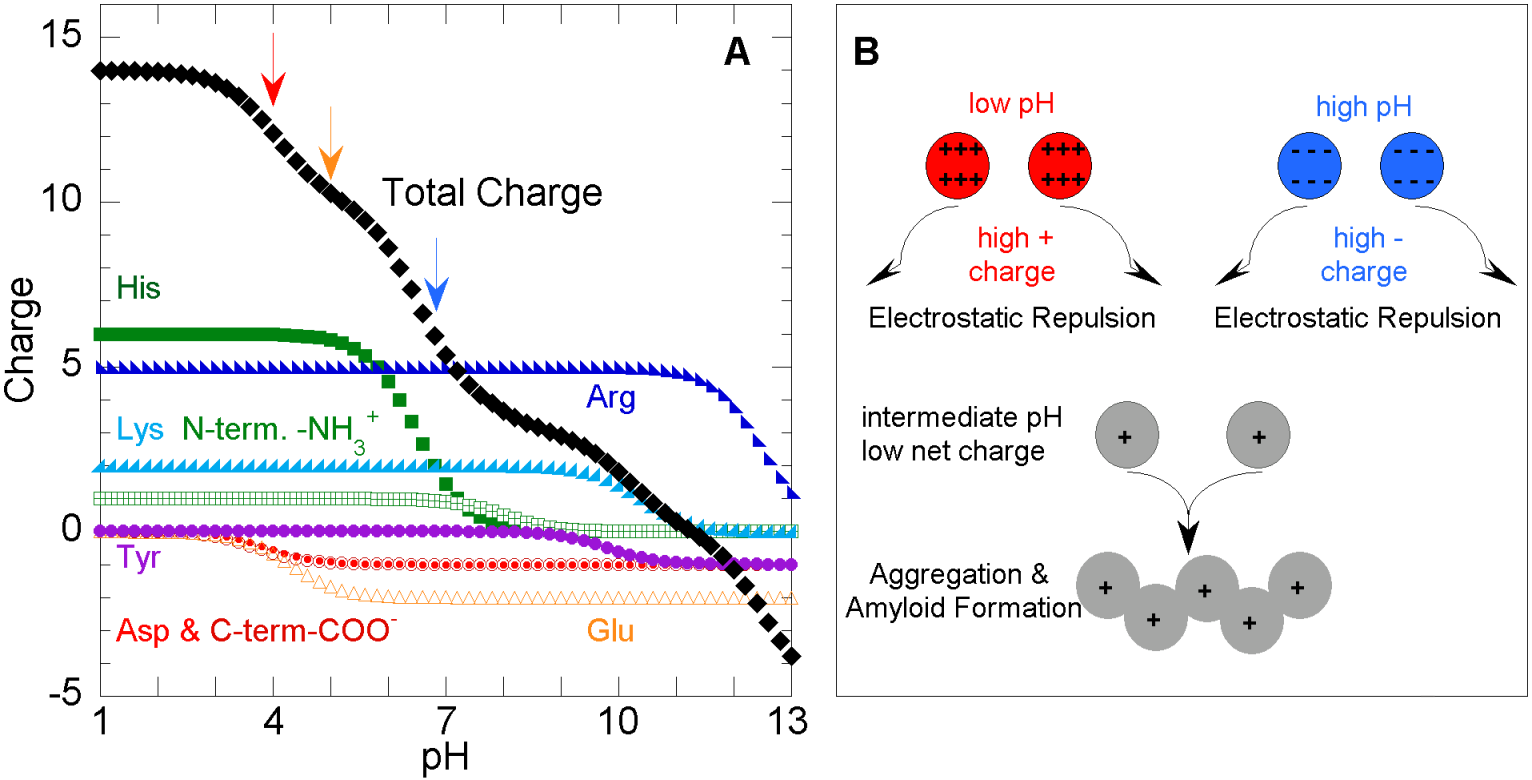
Electrostatic repulsion modulates the aggregation of TDP-43’s C-terminal domain. **A**. Calculated total net charge (black diamonds) on the TDP-43 C-terminal domain (CTD) as a function of pH. The contributions of different types of titratable groups are shown as colored symbols (C-terminal carboxylate = red open circles, Asp 406 = small red circles, Glu 271 and 362 = gold open triangles, His 264 and hexaHis tag = green squares, N-terminal amine = open crossed green squares, Tyr 374 = purple circles, Lys 263 and 408 = sky-blue triangles, and Arg 268, 272, 275, 293, and 361 = blue triangles. The red, gold, and blue arrows mark the net charge at pH 4 (+12.1), pH 5 (+10.3), and pH 6.8 (+6.0). The underlying data are shown in S1 Data, columns K–U. **B**. Model for the electrostatic control of amyloid formation. At pH extremes, polypeptides carry a high positive or negative net charge. The resulting unfavorable charge–charge interactions impede association. At intermediate pH values, the net charge is low and electrostatic repulsion is too weak to prevent oligomerization and amyloid formation, which is favored by strong hydrogen bond networks, hydrophobic interactions, or both.

Finally, regarding the pathological TDP-43 CTD variants studied, A315E would decrease the net charge over the pH range studied and Q331K would increase it. It is notable that A315E forms amyloid much faster than Q331K (see Figs 2 and 3 of [2]), as predicted by the charge-repulsion model. Considering that (i) aggressive Aβ mutations like “Arctic” (E22G), “Dutch” (E22Q), “Iowa” (D23N), and “Italian” (E22K) also decrease the net charge at physiological pH [8]; (ii) there is a correlation between higher net charge and slower kinetics of amyloid formation [10]; and (iii) charge–charge interactions modulate aggregation and amyloid formation in alpha synuclein [11], amylin [12], a β_2_-microglobin-derived peptide [13], huntingtin [14], superoxide dismutase [15], and Tau [16] and affect aggregation kinetics [11] and morphology [17], these results point to the general importance of electrostatic repulsion in governing the amyloid formation rather than H-bond formation as proposed by Lim, Wei, Lu, and Song [2].

## Supporting Information

S1 Data Supporting Data File 1. This file includes the data used to generate Figs 1 and 2A.
(XLSX)

## Abbreviations

ALS: amyotrophic lateral sclerosis
CD: circular dichroism
CTD: C-terminal domain
FTLD: frontotemporal lobar dementia
H-bond: hydrogen bond
mRNP: mRNA-protein
NMR: Nuclear Magnetic Resonance
